# Systemic Amyloid Light Chain Amyloidosis With Repeated Syncope Due to Severe Orthostatic Hypotension Caused by Autonomic Neuropathy: A Case Report

**DOI:** 10.7759/cureus.73320

**Published:** 2024-11-09

**Authors:** Yohei Mori, Tsuneaki Kenzaka

**Affiliations:** 1 Department of General Medicine, Mitaki General Hospital, Yokkaichi, JPN; 2 Department of Internal Medicine, Hyogo Prefectural Tamba Medical Center, Tanba, JPN; 3 Division of Community Medicine and Career Development, Kobe University Graduate School of Medicine, Kobe, JPN

**Keywords:** autonomic neuropathy, biopsy, cardiogenic syncope, eosinophilic amorphous, immunohistochemical staining, pulmonary thromboembolism

## Abstract

Amyloid light chain (AL) amyloidosis is a disease in which ALs, which are proteins with fibrous structures, are deposited in systemic organs, causing functional impairment. Diagnosis is often difficult because of non-specific and varied symptoms. We report a case of systemic AL amyloidosis that was diagnosed as a result of repeated syncope.

A 76-year-old woman was brought to the emergency room with multiple episodes of loss of consciousness over the past five years. She visited the major hospital, where pulmonary thromboembolism and symptomatic epilepsy were considered possible causes. Orthostatic hypotension was observed after being transferred to our hospital for rehabilitation. We performed diagnostic tests, including blood tests, imaging, and a head-up tilt test, which confirmed severe orthostatic hypotension. A gastrointestinal biopsy with Congo red staining confirmed the presence of amyloid deposits. AL amyloidosis (λ) was diagnosed using immunohistochemical staining. Given her age and prolonged bed rest, she was determined that she could not tolerate chemotherapy and was discharged upon her request.

To the best of our knowledge, this is the first report of systemic AL amyloidosis presenting with orthostatic hypotension severe enough to cause syncope due to autonomic neuropathy. Autonomic neuropathy should be considered, and amyloidosis should be included in the differential diagnosis when a patient presents with recurrent syncope.

## Introduction

Amyloid light chain (AL) amyloidosis is a medical condition in which ALs are deposited in various organs and tissues, leading to dysfunction. This condition is characterized by various non-specific symptoms, including congestive heart failure, diarrhea, fatigue, and weight loss [1,2]. The diagnosis of this condition can be challenging owing to its non-specific characteristics. It is worth noting that syncope, a rare occurrence of AL amyloidosis (immunoglobulin light chain amyloidosis), has been observed in certain cases [3]. Most cases of syncope are cardiogenic, often resulting from factors such as severe atrioventricular block, valvular disease, and low output [3,4].

Peripheral neuropathy is also observed in 17-35% of patients with AL amyloidosis [5], with complaints of sensory disturbances such as numbness in the hands and feet; motor disturbances including paralysis, dizziness, and sweating; and autonomic disturbances such as diarrhea and constipation [6]. When these symptoms represent the primary manifestation of the disease, diagnosis is reported to be delayed [7]. However, syncope due to severe autonomic neuropathy has rarely been reported [8,9]. Herein, we describe a case of severe orthostatic hypotension and recurrent fainting attacks due to autonomic neuropathy.

## Case presentation

A 76-year-old woman, having experienced multiple episodes of loss of consciousness over the past five years, underwent a comprehensive examination at another hospital; however, the cause was undetermined, and she remained under observation.

One winter morning, her family found her unconscious on her bed at home and rushed her to a major hospital. She had been experiencing dizziness and fatigue for over a month before she was transferred to the hospital. On arrival, she had impaired consciousness (eye-opening 1, verbal response 2, and motor response 4) and low blood pressure, which could not be evaluated. Her rectal temperature was 26.4°C, heart rate was irregular (33 beats/min), respiratory rate was 16 breaths/min, and peripheral capillary oxygen saturation (SpO2) was 97% (room air).

**Figure 1:**
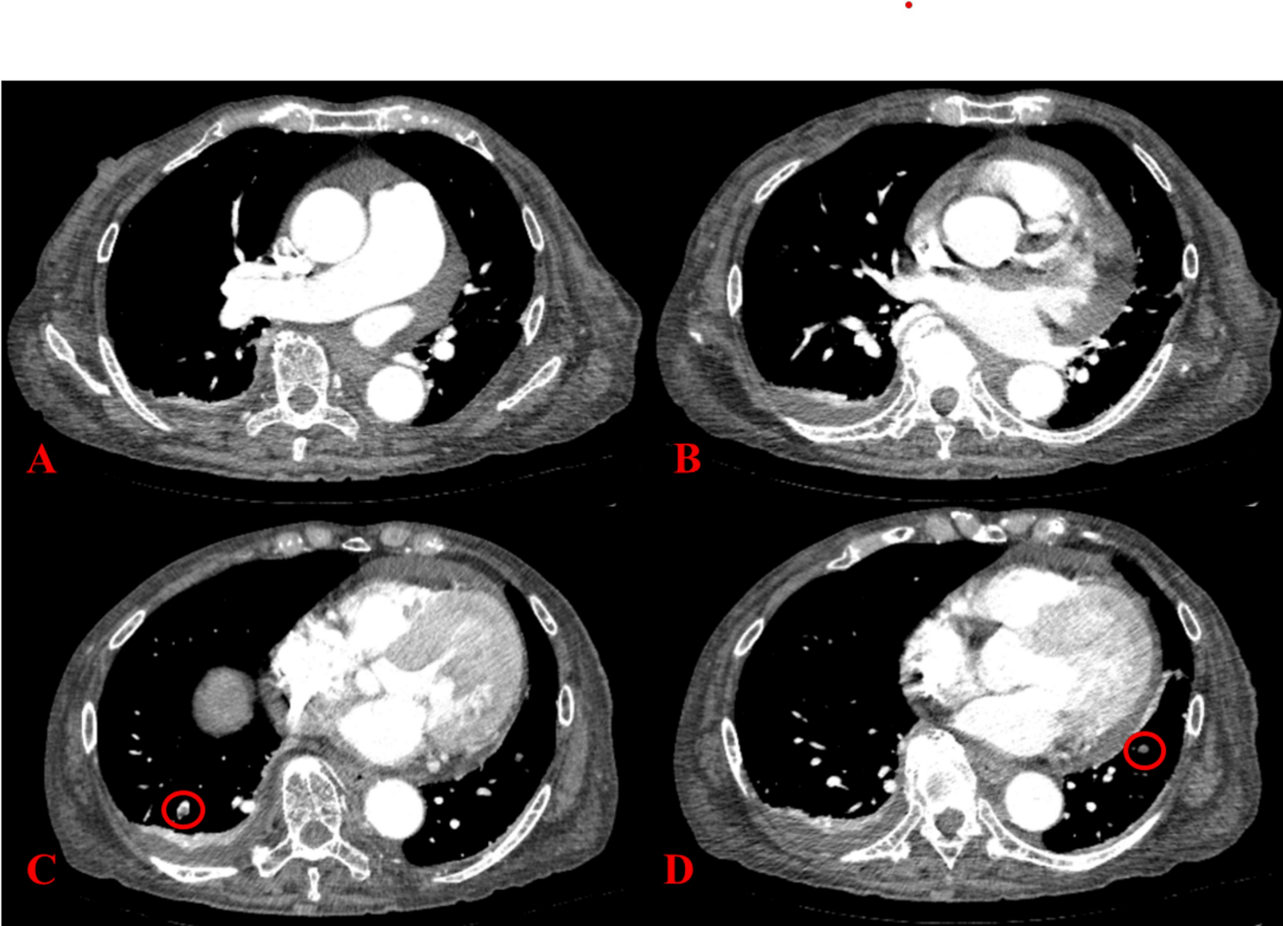
Findings of contrast-enhanced chest computed tomography Thrombi observed in the right (C) and left (D) basal pulmonary arteries. Because no thrombus was found in the main pulmonary arteries (A) (B) and no right ventricular dysfunction was identified, the diagnosis was low-risk pulmonary embolism.

In the high care unit (HCU), impaired consciousness, blood pressure, body temperature, and bradycardia improved with the rewarming and administration of vasopressors. The patient was discharged from the HCU on the fourth day of hospitalization. However, she continued to experience syncope after being discharged from the HCU, and chest contrast-enhanced computed tomography (CT) performed five days after hospitalization confirmed pulmonary thromboembolism (Figure 1).

Her pulmonary thromboembolism was treated with apixaban 10 mg b.i.d. for seven days, following which the dose was reduced to 5 mg b.i.d. Because of another syncope, epilepsy was suspected, and an electroencephalogram (EEG) examination was performed on the 28th day of admission. No epileptic waves were observed. However, given her history of stroke, the physician suspected symptomatic epilepsy and initiated levetiracetam 1000 mg b.i.d. Five days later, her previous attending physician determined that the syncope had resolved. She required rehabilitation for disuse syndrome due to prolonged bed rest and was transferred to our hospital 32 days after hospitalization.

Progress after admission to our hospital

At breakfast on the day after admission, she lost consciousness when she was moved from a supine to a sitting position with her head elevated on a motorized bed. She was hypotensive without tachycardia, with a blood pressure of 61/47 mmHg and a pulse rate of 68/min. Subsequently, the patient experienced repeated episodes of loss of consciousness, which occurred while standing or after eating. Additionally, we discovered that the patient had urinary retention, which remained unexplained by her prior physician.

Based on these symptoms, we considered the patient's condition to be autonomic neuropathy and conducted a thorough examination. We re-examined the patient's medical history and found that all episodes of syncope occurred immediately after standing or sitting. Before these episodes, the patient had visited a local clinic for hypertension and had previously experienced cerebral infarction. However, her antihypertensive medication had been discontinued a month earlier owing to a decrease in her blood pressure.

She had no history of smoking or alcohol consumption. The patient had no family history of amyloidosis or sudden cardiac death. Her body mass index was 22. Physical examination revealed no evidence of macroglossia. The lung sounds were normal. Heart sounds showed a Levine 3/6 pan-systolic murmur with the apex of the heart at the strongest point. Muscle strength and sensory function were normal. Tendon reflexes were preserved in all extremities. Central neurological findings showed no abnormalities, apparent Parkinsonism, or cognitive impairment. Blood tests revealed slight anemia. There was no evidence of thyroid dysfunction, adrenal insufficiency, glucose intolerance, symptomatic electrolytes, or vitamin abnormalities (Table 1).

**Table 1:** Laboratory findings Slight anemia was observed. Additionally, sodium, potassium, chloride, calcium, magnesium, and serum iron were all mildly low. Ferritin and vitamin B12 were elevated.

Parameter	Recorded value	Standard value
White blood cell count	3,900/µL	3,300–8,600/µL
Hemoglobin	10.2 g/dL	11.5–15.0 g/dL
Mean corpuscular volume	96.1 fL	80–99 fL
Mean corpuscular hemoglobin	32.8 pg	25.0–34.0 pg
Mean corpuscular hemoglobin concentration	34.1%	31.0–36.9%
Platelet count	21.6×10^4^/µL	15–35×10^3^/µL
Erythrocyte sedimentation rate	14 mm/h	≥15 mm/h
C-reactive protein	0.1 mg/dL	≤0.14 mg/dL
Total protein	5.2 g/dL	6.6–8.1 g/dL
Albumin	2.7 g/dL	4.1–5.1 g/dL
Total bilirubin	1.3 mg/dL	0.4–1.5 mg/dL
Aspartate aminotransferase	11 U/L	13–30 U/L
Alanine aminotransferase	8 U/L	7–23 U/L
Lactase dehydrogenase	191 U/L	124–222 U/L
γ-glutamyl transpeptidase	17 U/L	13–64 U/L
Creatine kinase	30 U/L	41–153 U/L
Blood urea nitrogen	12.4 mg/dL	8–20 mg/dL
Creatinine	0.65 mg/dL	0.46–0.79 mg/dL
Sodium	137 mEq/L	138–145 mEq/L
Potassium	3.3 mEq/L	3.6–4.8 mEq/L
Chloride	100 mEq/L	101–108 mEq/L
Calcium	8.6 mg/dL	8.8–10.4 mg/dL
Phosphorus	3.2 mg/dL	2.5–4.5 mg/dL
Magnesium	1.7 mg/dL	1.8–2.6 mg/dL
Iron	42 μg/dL	48–154 μg/dL
Ferritin	233.9 ng/dL	6.4–167.1 ng/dL
Glucose	97mg/dL	73–109 mg/dL
Hemoglobin A1c	5.7%	4.9–6.0%
Vitamin B12	1,219 pg/mL	180–914 pg/mL
Folic acid	4.0 ng/mL	≥4.0 ng/mL
Thyroid stimulating hormone	1.11 μIU/L	0.34–4.22 μIU/L
Free T4	1.73 ng/dL	0.77–1.74 ng/dL
Adrenocorticotropic hormone	28.5 pg/mL	7.2–63.3 pg/mL
Cortisol	11.8 μg/dL	3.7–19.4 μg/dL
Plasma aldosterone concentration	11.1 pg/mL	4.0–82.1 pg/mL
Plasma renin activity	0.2 ng/mL/h	0.2–2.3 ng/mL/h
Antinuclear antibody	Negative	Negative
anti-Ro/SSA	Negative	Negative
anti-Ro/SSB	Negative	Negative
Carcinoembryonic antigen	1.9 ng/mL	0.1–5.0 ng/mL
Carbohydrate antigen 19-9	55.3 U/mL	≤37 U/mL
Soluble interleukin 2 receptor	366.0 U/mL	122–496 U/mL
Angiotensin converting enzyme	9.1 IU/L	7.0–25.0 IU/L
Prothrombin time/International normalized ratio	1.30	0.80–1.20
Activated partial thromboplastin time	37.2 s	26.9–38.1 s
Urinalysis		
Protein	9.3 mg/dL	<5mg/dL
Creatinine	28.0 mg/dL	100.0–150.0mg/dL
Fractional excretion of urea nitrogen	40.0%	40–60%
Fractional excretion of sodium	0.6％	1–2%
Fractional excretion of potassium	5.0%	10–20%

**Figure 2:**
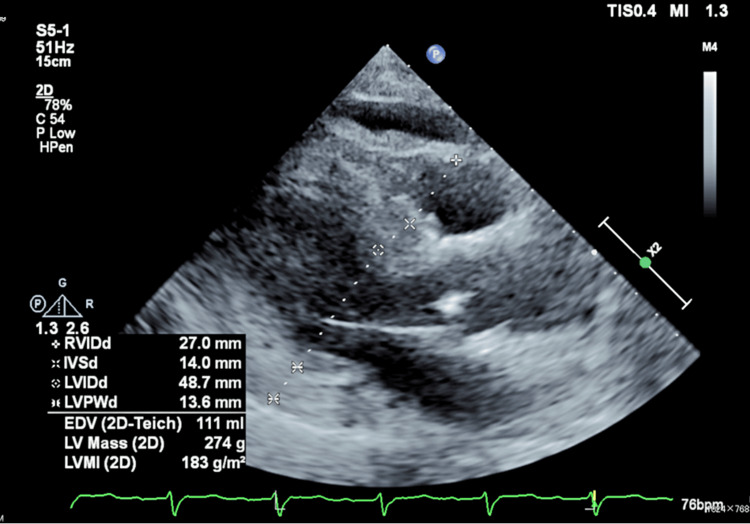
Findings of transthoracic echocardiography Left ventricular hypertrophy can be seen with interventricular septal end diastole (IVSd) of 14.0 mm and left ventricular posterior wall end diastole (LVPWd) of 13.6 mm. Right ventricular internal dimension diastole (RVIDd) was 27.0 mm. Left ventricular internal dimension diastole (LVIDd) was 48.7 mm. End-diastolic volume (EDV) was 111 mL. Left ventricular (LV) mass was 274 g. LV mass index (LVMI) was 183 g/m^2^.

Transthoracic echocardiography revealed concentric hypertrophy of the left ventricle and mild mitral regurgitation (Figure 2). However, no systolic or diastolic dysfunction could explain the syncope (ejection fraction 64% (≥50%), stroke volume 72 mL (60-130 mL), E/e' ratio 29.9 (<14), and septal-e' 3.48 cm/s, lateral-e' 2.32 cm/s). Systolic anterior movement, left ventricular outflow tract obstruction, or asymmetric septal hypertrophy were not observed.

**Figure 3:**
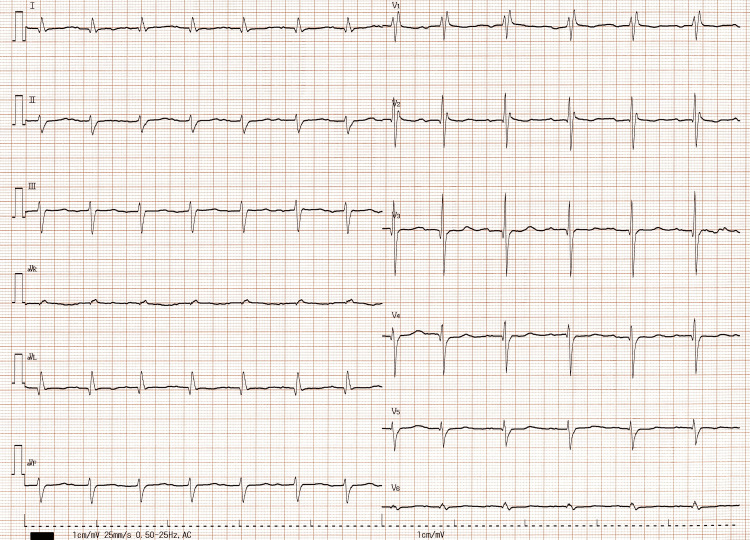
Resting electrocardiography A complete right bundle branch block and prolonged P-R interval can be observed. Electrical axis was -68°.

A 24-h Holter electrocardiogram (ECG) and resting ECG showed only a complete right bundle branch block and supraventricular extrasystoles, and no arrhythmias that could cause syncope (Figure 3) were observed.

CT scans of the chest, abdomen, and pelvis revealed no suggestive cardiovascular disease, malignant disease, or other notable findings. Cranial magnetic resonance imaging revealed ischemic changes only in the cerebral white matter. Spinal magnetic resonance imaging revealed no notable findings.

The head-up tilt test was performed to evaluate orthostatic hypotension. In the resting supine position, her blood pressure was 112/75 mmHg, and her pulse was 80 bpm; however, when the tilt angle was elevated to 55°, she lost consciousness, her blood pressure was 40/unmeasurable, and her pulse was 78 bpm. When the tilt angle was reduced to 30°, the patient immediately regained consciousness, with a blood pressure of 88/70 mmHg and a pulse of 88 bpm. These results indicate severe orthostatic hypotension.

**Figure 4:**
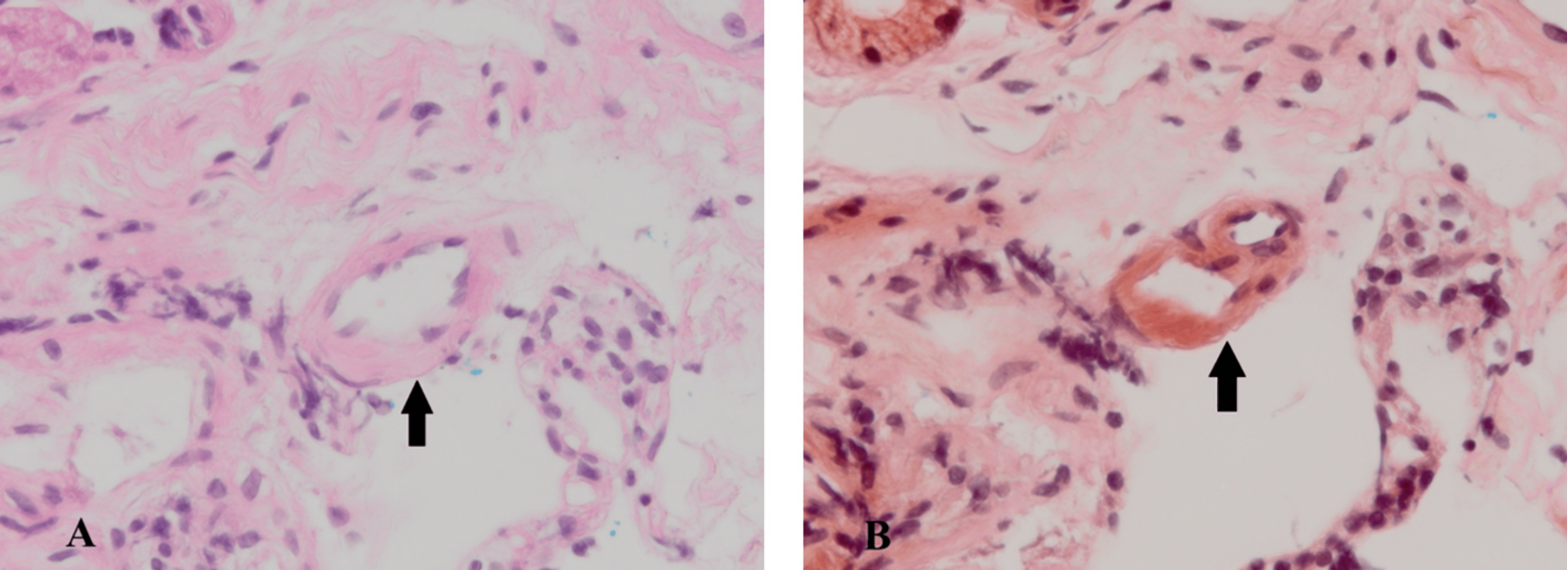
Histological image of the duodenal bulb (Congo red staining, magnification 40×) Hematoxylin & Eosin staining reveals eosin-stained areas that are observed as homogeneous structureless (A), whereas Congo red staining reveals amyloid deposition areas that are stained red (B).

Upper gastrointestinal endoscopy was performed for the workup of paraneoplastic syndrome and histologic evaluation of amyloidosis, and biopsies were obtained from the greater curvature of the gastric antrum, greater curvature of the gastric body, and duodenal bulb. Deposition of eosinophilic amorphous matter was confirmed in the submucosal vascular wall of the duodenal bulb with positive Congo red staining (Figure 4). AL amyloidosis (λ) was diagnosed using immunohistochemical staining.​​​​​

Differential diagnosis

Syncope can be classified as cardiogenic, neurally mediated, or orthostatic hypotension [10]. Cardiogenic shock is caused by arrhythmias, structural cardiac abnormalities, cardiopulmonary abnormalities, and abnormalities of the great vessels [10]. Neurally mediated syncope is caused by vasovagal reflex, situational syncope, and carotid sinus syndrome [10]. Orthostatic hypotension is caused by drug-induced hypovolemia and autonomic neuropathy [10].

In this case, echocardiography and a 24-h Holter ECG ruled out cardiogenic causes. Additionally, the possibility of neurally mediated syncope was ruled out based on the circumstances of the syncope. Therefore, we investigated orthostatic hypotension. The patient was not taking any medications that could cause hypotension. She had good oral intake with no diarrhea, vomiting, or bleeding, which ruled out fluid deprivation.

Therefore, we investigated the diseases that cause autonomic neuropathy. The following etiologies were further investigated: Parkinson's disease, dementia with Lewy bodies, endocrine disorders such as diabetes mellitus, spinal cord injury, autoimmune diseases, paraneoplastic syndrome, sarcoidosis, and amyloidosis [10]. Subsequently, amyloidosis was diagnosed based on biopsy examination results from the duodenal bulb obtained using upper gastrointestinal endoscopy after investigating these diseases in parallel.

Course after diagnosis

In parallel with various tests, she was treated for orthostatic hypotension. First, salt loading at the rate of 13 g/day and the use of elastic stockings were attempted; however, no improvement was observed. Subsequently, amedinium methyl sulfate 20 mg b.i.d. and midodrine hydrochloride 4 mg b.i.d. were administered sequentially, followed by fludrocortisone 0.1 mg once daily. However, the patient's condition remained unchanged.

On day 27, after admission, a diagnosis of systemic amyloidosis was made. On day 32, she was transferred from our hospital to the major hospital to be treated by a neurologist and hematologist.

After the transfer, she underwent further examinations and was considered for treatment. However, due to her advanced age and weakness from prolonged bed rest, her physicians determined that she could not tolerate chemotherapy. She was discharged home after two months of readmission to the major hospital, as she preferred to stay at home under her family’s care if no treatment was to be administered.

## Discussion

The frequency of orthostatic hypotension in systemic AL amyloidosis is approximately 14-30% [1,11]. Orthostatic hypotension caused by autonomic neuropathy is a common symptom of systemic AL amyloidosis; it includes dizziness and lightheadedness, and a few reports have mentioned syncope [2,8,9,12].

In contrast, it has been reported that 14-20% of cases complicated by cardiac amyloidosis experience syncope [3,13]. Most reports of syncope leading to a diagnosis of systemic AL amyloidosis have been cardiogenic, such as valvular disease and low cardiac output [3, 4]. Furthermore, there have been reports of syncope due to arrhythmias or severe atrioventricular block [3].

The present case showed abnormal echocardiographic structural findings suggestive of concomitant cardiac amyloidosis, such as concentric hypertrophy of the left ventricle and mild mitral regurgitation. However, no abnormal functional findings suggestive of syncope were observed, and no arrhythmias or severe atrioventricular block were observed on Holter ECG.

Pulmonary thromboembolism, which impeded the final diagnosis by the previous physician, presents with syncope. The incidence of thromboembolism in systemic amyloidosis is estimated to be 5-10% [14]. The pulmonary thromboembolism that developed in the present case was associated with amyloidosis. However, there was no evidence of severe right ventricular dysfunction or hemodynamic abnormalities that would strongly suggest a cause of syncope. 

To our knowledge, there are few reports of systemic AL amyloidosis with orthostatic hypotension due to autonomic dysfunction severe enough to cause syncope, especially in the absence of abnormal findings suggestive of cardiogenic syncope, as seen in the present case [8,9,12].

The present case had abnormal echocardiographic findings, pulmonary thromboembolism, and symptomatic epilepsy; however, there were no abnormalities causing syncope. Consequently, the diagnosis of systemic amyloidosis took time. More than five years have passed since the initial syncope to the confirmation of the diagnosis. The patient had been symptomatic for two months prior to admission to our hospital, and the disease remained undiagnosed during 32 days of admission to the previous major hospital.

In addition to chemotherapy, new treatment methods for amyloidosis, including the use of molecular-targeted agents for the treatment of multiple myeloma, have been established [15], and prolonged survival has been reported [16]. Therefore, early diagnosis of amyloidosis can lead to a good prognosis. It has been suggested that autonomic neuropathy may lead to an early diagnosis of amyloidosis [12]. Therefore, patients with syncope should be interviewed in detail and their symptoms carefully assessed for possible autonomic neuropathy.

## Conclusions

We present a case of systemic AL amyloidosis diagnosed as a result of repeated syncope. This is the first report of systemic AL amyloidosis presenting with orthostatic hypotension that is severe enough to cause syncope due to autonomic neuropathy. Despite the availability of effective therapies, AL amyloidosis remains a diagnostic challenge because of its nonspecific and varied symptoms. Thus, autonomic neuropathy should be considered a possible cause of recurrent syncope, and amyloidosis should be included in the differential diagnosis.
